# Black Healers, Surgeons and ‘Witches’: Medicine, Mobility and Knowledge Exchange in Swedish St Barthélemy 1785–1815

**DOI:** 10.1093/shm/hkaa092

**Published:** 2021-08-03

**Authors:** Fredrik Thomasson

**Keywords:** Obeah, Afro-Caribbean medicine, Black healers, Swedish colonialism, colonial medicinal legislation

## Abstract

When Swedish civil servants took possession of the Caribbean island of St Barthélemy in 1785, they discovered a complex medical landscape in which Black healers played important roles. They competed with white physicians for patients and formed an itinerant community—both voluntary and forced in nature—which travelled throughout the archipelago exchanging remedies and practices. The healers’ work was not associated to revolt and rebellion as in many other Caribbean territories and the Swedish court of law treated them with less cruelty than in many other colonies. The healers’ activities cannot be simply reduced to acts of resistance to slavery; many of them gained the trust of large parts of both Black and white communities. Their interactions with people on the surrounding islands show how Caribbean colonial historiography gains from a wider geographical contextualisation, allowing a better understanding of the Black population’s role in healing and medicine.

##  

When the first Swedish colonial administrators took possession of the small Antillean island of St Barthélemy in March 1785, they discovered a complex medical landscape in which Black healers played important though contested roles.^1^

A French report written just before the Swedish takeover stated that ‘a negro slave serves as the island’s doctor, everyone trusts him and it is said that he is skilled in blood-letting, and with fractures and wounds’. The enslaved man Coq d’Inde was listed in a Swedish census as a 70-year-old *chirurgien*, and there are no indications that he was prohibited from practising.^2^ Equal indulgence was not afforded to another enslaved man who, in a petition in May the same year was accused—‘on behalf of all inhabitants’—of being a poisoner. The governor then expelled the ‘negro named Charles [who] is a poisoner ... and so as to prevent disagreeable accidents that the presence of such an evil man may result in, he cannot be allowed to stay on the island’.^3^

The accusation of poisoning was intimately connected to the French-speaking white population’s fear of the Black population, and this particular case had perhaps been the result of Charles administering traditional medicine. It was nominally illegal throughout the Caribbean for Black people to treat physical ailments, but there is much evidence that not only Black inhabitants, but also white communities, sometimes including physicians and colonial administrators, made use of and appreciated the services of these healers.

The range of services offered by these Black healers in St Barthélemy was extensive. Coq d’Inde bled white patients who in turn contracted Black healers to treat their slaves and used Black enslaved midwives to deliver their own children. When both white physicians and Black healers had failed to cure the white merchant Bernard Lion’s slaves, he enlisted the services of the enslaved woman Rosalie who possessed an all-seeing eye, she is the ‘witch’ referred to in the article’s title. I avoid clearly demarcating differences between magical and medicinal practices, both because such differences in many cases are impossible to identify, and because such distinctions in themselves are ahistorical in several of the cases analysed here.

The newly arrived officials did not assimilate the association between Black healers and witchcraft, poisoning and rebellion that was common throughout the Caribbean. The Swedish response to these activities did not correspond to the often cruel punishments meted out by other colonial justice systems. One of the particularities of Swedish governance is that no special court proceedings were held for the enslaved population and that, unlike in the French and British Caribbean, general court protocols contain their detailed testimonies and accounts.^4^ The Swedish cases presented here thus form interesting contributions to the knowledge of the activities of Antillean healers and offer an insight into healing practices that are more difficult to follow on many other islands.

## Forced and Voluntary Mobility

The work of Black healers in St Barthélemy highlights several issues discussed in the vast literature on what Londa Schiebinger calls the Atlantic world medical complex. Schiebinger questions whether ‘invisible boundaries of empire limit interisland intellectual exchange’.^5^ The contention here is that such boundaries are even harder to perceive among the islands that form the Lesser Antilles. The forced and voluntary mobility of Black healers created an archipelago-wide network, through those who offered their services in St Barthélemy and surrounding islands. Pablo F. Gómez’s description of an ‘early modern Caribbean healing marketplace’ is an apt designation of what can be described as both an exchange of cures and practices, but also of fears and rumours, given that some accusations of poisoning and witchcraft were likely aimed at closing down rival practitioners.^6^ Although economic exchanges did occur in such a marketplace, it should not be understood as purely economic in nature. The market metaphor also captures the exchange of knowledge, methods and clients among competing healers. In St Barthélemy, white physicians vied with Black healers to gain both the trust of, and money from, a diverse group of clients.^7^ The marketplace concept’s main weakness is that it gives a semblance of equality between actors. Never did such parity exist, Black healers faced restrictions unknown to the white community.

**Fig. 1:**
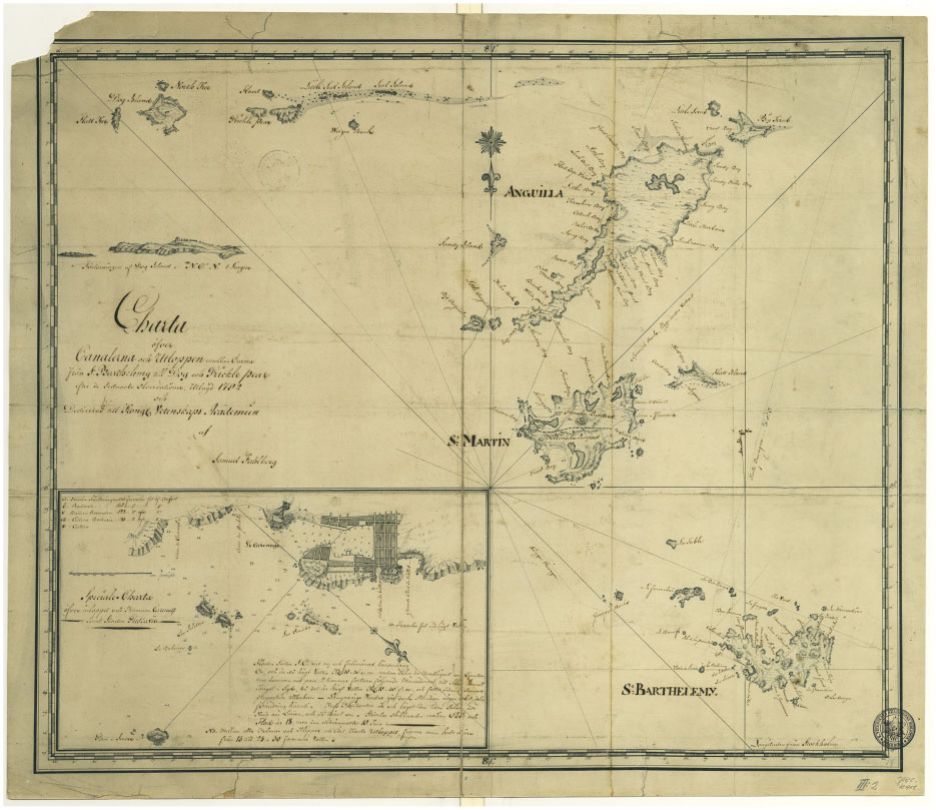
The 1792 hand-drawn map by Government Doctor Samuel Fahlberg of St Barthélemy, St Martin and Anguilla, lower left insert is a plan of the recently built town of Gustavia. The Center for History of Science, Royal Swedish Academy of Sciences, Stockholm.

Exchange and mobility are intimately connected to how we conceptualise Caribbean history and geography. The Swedish island lay close to territories belonging to several major European colonial powers. A 1792 hand-drawn map of St Barthélemy sets the island in its archipelagic context (Figure 1). Not only can these islands depicted on the map be seen from St Barthélemy, St Martin—still divided between France and the Netherlands—and the British territory of Anguilla, but so can St Kitts, Nevis, Sint Eustatius, Saba, and, on clear days, even Montserrat is visible.

Caribbean colonial historiography has traditionally been framed in predominately national narratives, often giving precedence to the colony’s relationship to the metropole.^8^ However, it is fruitless to frame the history of St Barthélemy in such a perspective. There are particularities to Swedish rule in St Barthélemy—and one is the relative leniency towards the Black healers discussed here—but such traits are the result of the merging of metropolitan mindsets and the Caribbean context, rather than expressions of direct rule from Sweden.^9^

## Importing and Adapting Colonial Medical Legislation

At the time of the Swedish takeover, in 1785, St Barthélemy comprised c. 460 white, 280 enslaved, and ten free Black inhabitants. It was immediately understood that the island was too small and arid to support any large-scale agriculture and the island was declared a free port in 1786. The Swedish administrators did their utmost to attract commerce and shipping. Following the slave rebellions and the wars arising out of the French Revolution the island began to attract a large number of immigrants. During the first decades of Swedish rule, the population grew to 5,000–6,000 people. The island’s capital, Gustavia, became a bustling port, comprising a cosmopolitan mixed society, with a c. 70 per cent Black majority.

The governor of St Barthélemy had extensive executive and legislative powers. In 1785 when he expelled the alleged enslaved poisoner Charles the island had yet to see a court of law and there was no colonial law which supported such decisions. The Swedish administrators soon realised that the territory needed a slave law and in June 1787 the *Ordinance concerning the Treatment & Police of Negroes & Coloured People* was simultaneously proclaimed in French and English. This first comprehensive Swedish slave law was a modified version of a 1783 French *code noir* ordinance. It retained the French original’s concern with the regulation of medicine and the fear of poisoning.^10^ Article 6 stipulated that:

No Negroes or Couloured (*sic*) people of any kind whatsoever, be they either free or slaves, shall be in any measure permitted to practise medicine or surgery, nor make any preparations for sick people ... under what pretext soever ... under a penalty of five hundred Livres for the first time, against the free persons, and the second time corporal punishment and slaves shall be condemned to Chains, and the Master loose (*sic*) the price or value of said slave; not having prevented him.^11^

Article 7 was an abbreviation of the original French law that aimed ‘to stop a disorder that ruins many planters’^12^:

All Persons who have the knowledge, in their vicinity or elsewhere, of any negroes or other slaves publickly suspected to be poisoners or distributers of drugs shall make it known to Government, that the Guilty may be severely punished.

Thus, the St Barthélemy law prohibited all Black inhabitants—both enslaved and free—from practising and distributing medicine, but did not make explicit references to religious or allegedly magical practices.^13^

## Obeah and Rebellion

The Swedish response to the activities of Black healers in St Barthélemy was distinct from that of other colonial powers with longer histories in the Antilles. References to Black people’s spiritual and medical practices appear in academic studies under the broad labels of *Obeah* in the British domains, while *empoisonnement* [poisoning] and *Vodou* are used in the French Caribbean. Defining these terms has always been a challenge: the meaning of Obeah changed over several centuries and in the French colonial context one of the main issues is how ‘poisoning’ became related to magic and how the fear of such acts turned into panic.^14^

Although witchcraft was decriminalised in France in 1682 and in Britain in 1736, these reforms did not include the colonies. The increasing divergence between metropolitan and colonial legislation and practice, and how Obeah and ‘poisoning’ were used in the construction of ideas of racial superiority, is an important theme in the academic literature.^15^ During the second half of the eighteenth century, the allegedly magical practices of Black populations in the French and British colonies became associated with the fear of rebellion and increasingly strict legislation was implemented throughout the archipelago.^16^ Jonathan Dalby concludes that, on Jamaica from 1760 until the abolition of slavery in 1834, ‘in terms of perceived seriousness – as suggested by severity of punishment – Obeah was behind only homicide, attempted homicide, and rebellion.’^17^ Famous episodes, such as the 1750s Makandal case on Saint-Domingue, are well known for the gruesome punishments they entailed. Courts on Martinique tried and executed slaves for poisoning with little regard to judicial procedure long into the nineteenth century.^18^ Danish Caribbean courts used torture to produce evidence and inflicted corporal punishments on the enslaved who were accused of witchcraft and Obeah.^19^

The Swedish administrators rarely referred to the Black population in St Barthélemy as threatening, but they did often worry about the consequences of uprisings in other colonies. They might have been right in assuming that the risk of rebellion in St Barthélemy itself was low.^20^ The island could not sustain large scale agriculture and the worst cruelties of the plantation system were not present, and while the majority of the population was Black, the free Black group was also unusually large. In 1806, Gustavia’s population comprised 1,424 enslaved, 802 free Black and 833 white persons.^21^

Rebellion was not explicitly associated with magical practices in St Barthélemy and instead of adopting Caribbean conceptual links between healing, magic, revolt and danger, the Swedish colonial administrators’ thinking was shaped by changes in the metropole’s attitude to witchcraft. King Gustav III declared in 1778 that witchcraft was ‘a fanciful crime derived from papist delusions’.^22^ Although witchcraft was punishable by death until the criminal charge was abolished in 1779, no executions were carried out after 1704.^23^

Instead, magical practices in St Barthélemy were recognised as similar to those in Sweden where a wide range of medical practitioners supplied remedies and rituals.^24^ Colonial officials operated in the knowledge of the extensive Swedish legislation concerning medicine. Although royal decrees spelled out the main rules, the sector was largely managed by the Stockholm *Collegium medicum*. Such legislation was in principle valid on St Barthélemy, and sometimes referred to in protocols and sentences. However, Caribbean conditions were so different from the domestic ones that metropolitan legislation—and this was true for many areas of law—was often considered inapplicable or unenforceable. Yet, metropolitan laws that were considered especially important in the local context were sometimes adapted and proclaimed on the island, as seen in the case of the 11 November 1800 regulation that required all ‘médecins et chirurgiens’ to hold a licence.^25^ The legal environment was thus quite different on Swedish St Barthélemy than on many of the surrounding islands.

## ‘The Witch of St. Martins’

An 1806 court case serves to illustrate how the Swedish authorities responded to medical matters, while also revealing the forced mobility of Black healers who not only competed with each other but also with the white physicians working on St Barthélemy. The case also reveals widespread trust in Black healers’ medical and magical capabilities, and the court’s attempt to eradicate such beliefs.

In August, the white French-speaking merchant Bernard Lion submitted a petition accusing two enslaved men, Jean-Baptiste and Gabriel, of poisoning his slaves. During the previous year and a half, four of Lion’s slaves had died: Antoine and Maria of dropsy, Hyppolite after spitting blood for a long period, and a three-month-old baby of unknown causes. In addition to these deaths Jacques had gone mad, and Catherine suffered seizures. Despite consulting several physicians on the island no one had been able to cure Lion’s slaves. He had then turned to Jean-Baptiste, an enslaved healer born in Africa, who also failed. Lion became increasingly desperate and was told that there was a woman with exceptional powers on the neighbouring island of St Martin.

**Fig. 2:**
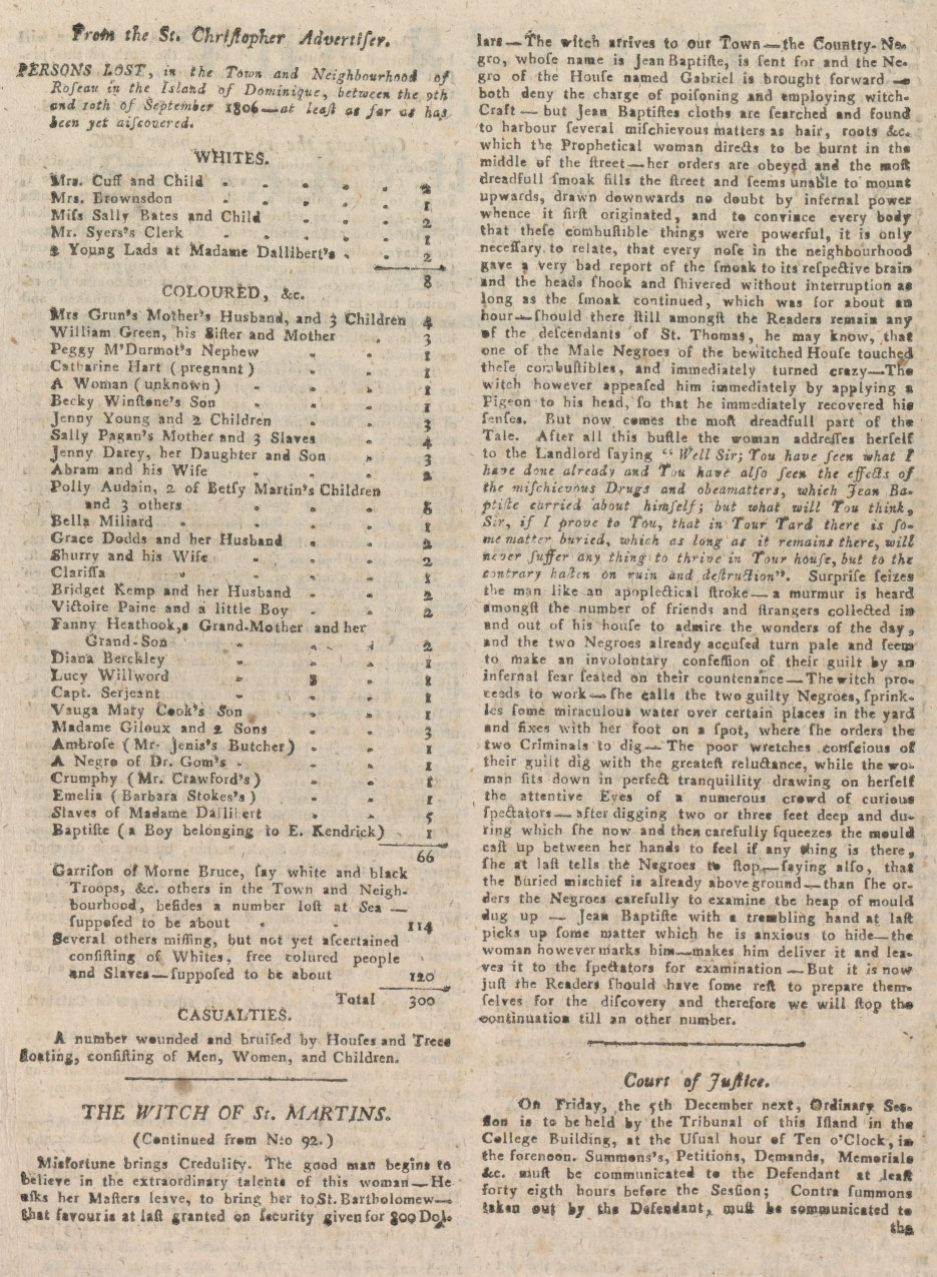
One of the instalments of *The Witch of St. Martins* articles. *The Report of St. Bartholomew*, No. 94, 15 November 1806. Uppsala University Library.

The events were not only documented in the ensuing court case, but also described in a series of articles entitled ‘The Witch of St. Martins’ published in the island’s newspaper, *The Report of St. Bartholomew* (Figure 2). The weekly newspaper was edited by Anders Bergstedt, who was also the island’s judge at this time. The first article was published two days after the court proceedings were initiated. Bergstedt probably wrote the accounts himself. The articles are rich in detail and ironical in their description of the events.

Bernard Lion followed the advice he was given and went to St Martin to meet Rosalie. Lion explained his problem and Rosalie, after using a magical mirror to investigate the matter, told Lion that *‘*Your Negroes can never thrive; part of them are poisened (*sic*); but independent of that, Your House & Yard are bewitched. The Authors are two Negroes, which my Glass exhibits to me, one is Your Own Negro and the other is a Coast Negro living in the North part of the Island’.^26^ Lion paid 800 Spanish dollars as security to Rosalie’s owner and brought her to St Barthélemy. This was around four times the normal price of a female slave and an indication that her owner profited from selling her services.

When Lion and Rosalie arrived on St Barthélemy, Lion summoned his slave Gabriel, whom Rosalie had referred to, as well as his friend the ‘Coast Negro’—that is born in Africa—Jean-Baptiste. Jean-Baptiste and Gabriel denied any involvement in poisoning or witchcraft, but Jean-Baptiste’s clothes were searched and ‘found to harbour several mischievous matters as hair, roots &c’. Rosalie gave these objects to another of Lion’s slaves who put them in his pocket and immediately started acting strangely. She put a live pigeon on his head which with some food and rum calmed him down, and then burned the objects in the street. Rosalie told Lion that in addition to the ‘mischievous Drugs and obeamatters’ found on Jean-Baptiste there were objects buried in Lion’s yard that would ‘hasten on ruin and destruction’. At this accusation, ‘the two Negroes already accused turn pale and seem to make an involontary (*sic*) confession of their guilt by an infernal fear seated on their countenance’.

Rosalie ordered the two slaves to dig in Lion’s yard, close to the back door. After digging the hole Rosalie ordered Jean-Baptiste to go through the upturned earth and ‘Jean Baptiste with a trembling hand at last picks up some matter which he is anxious to hide – the woman however marks him – makes him deliver it and leaves it to the spectators for examination’. One of the onlookers managed to confiscate some of the objects in the linen bundle found in the earth heap, and delivered them to the court for inspection.^27^

## Prosecuting Fraud instead of Magic

Following Bernard Lion’s petition to the court a legal case was opened the day after the yard had been dug up. The protocols give a detailed account of the events, often reproducing witness statements as direct speech. Such records must, of course, be read with circumspection. The historian Gabriel Debien commented upon French official sources and warned: ‘To describe the life of slaves using these sources is a paradox. It is never they who speak, who give testimony, but rather the managers and the masters who are white and refer to themselves as colonisers … One must always remember that the whites have the word.’^28^ Even though the Swedish records have been edited by court secretaries, what is recorded as direct speech and summaries of testimonies often provides detailed accounts of events and the positions of enslaved defendants or witnesses. As a comparison Sophie White makes a convincing case for that enslaveds’ depositions in the mid-eighteenth century courts of French Louisiana are recorded with accuracy and that they give us extraordinary insights into the lives of both the Black and indigenous population.^29^

A witness underlined that when Rosalie performed her acts in the street the language used was ‘termes nègres ou crëole’.^30^ French, English and respective Creoles were the main languages on the island, while Swedish was used by the authorities. In most of the cases discussed here, several languages were used. Such multilingual practice may have been a more important factor in Caribbean justice than has hitherto been recognised.

The protocols of these particular proceedings is an interesting case in point as the deliberations are mainly in Swedish. Depositions were translated into Swedish and the terms used for the alleged crimes were already attuned to the court’s position that the main crime in the case was not ‘poisoning’, instead the Swedish terms used by the court singled out Rosalie’s fraudulent behaviour and Jean-Baptiste’s medicinal work.

The court began by stating the reasons for investigating Jean-Baptiste, Gabriel and Rosalie’s cures and rituals: ‘Far from wanting to strengthen less enlightened persons’ already too deeply rooted prejudices and superstitions, [the Court] intends through a well-intentioned investigation to evince the shameless fraud, on one hand, and the inexcusable credulity, on the other hand, that are the causes of [Bernard Lion’s] present petition.’^31^

Lion’s petition requested that the court imprison Gabriel and Jean-Baptiste and accused them of ‘administering harmful herbal simples and other substances’, in other words, of poisoning his slaves.^32^ A constable arrested the slaves, including Rosalie whom Lion had not mentioned in the petition. As the case developed, it became clear that Lion’s petition would not deliver the expected outcome from an Antillean court. Instead of severely punishing the imprisoned slaves, they ended up being acquitted by the court and Lion himself was criticised for using Black healers and resorting to magic instead of using the island’s licensed physicians.

The court started by interrogating Rosalie. She told the court that she was c. 25 years old and had been born in Cayenne in French Guiana. A free Black merchant had bought her in Cayenne and sold her at auction in Gustavia. She had only been sold to the planter on St Martin eight months earlier. This explains how she already knew about Jean-Baptiste and Gabriel. The court demanded to see the mirror mentioned in the newspaper report, and concluded that it was of ‘the lowest quality possible, with a paper frame’.^33^ Rosalie was asked to show the court what she saw in the mirror but responded that the mirror only allowed her to see the culprits.

Jean-Baptiste was interrogated next. He was born in Africa, of the Nago (Yoruba) nation, and had been on St Barthélemy for 18 years. He was known as a ‘potion mixer’, that is, for dispensing traditional medicine, and enjoyed a good reputation on the island.^34^ It turned out that Lion had, on several previous occasions, paid Jean-Baptiste to cure Catherine and other slaves, among them Congo—whose name implies he was African-born—and Nicholas for ‘insanity’.^35^ Jean-Baptiste admitted that he had given Lion’s slaves potions both to drink and to use externally. Catherine was called and told the court that she had felt worse after Jean-Baptiste had treated her. Jean-Baptiste countered that the potions he had administered could not have been harmful given that ‘as was the custom of the negroes he had always drunk them together with the sick’.^36^ He added that if he had not been approached by Lion, he would never have concerned himself with the sick slaves. He denied all knowledge of the objects found in Lion’s yard and said he knew nothing about ‘evil arts’.^37^ The court ordered a search of his small house at his owner’s property in the countryside, but no medicinal substances were found. Gabriel—born on Guadeloupe—only seemed to have been implicated in the affair because he spent a lot of time with Jean-Baptiste. No witness had seen him prepare or administer medicine. That Gabriel was Lion’s slave is another indication that Lion took Rosalie’s accusations seriously as he risked forfeiting his property as stipulated in the 1787 slave law.

The bundle that Rosalie claimed to have found in the hole contained something that appeared to be a child’s umbilical cord, a small egg, Gumby beads, gunpowder, human hair and fruits, all kept in a calabash mixed with rum and other liquids and wrapped up in linen. Similar objects were often referred to as ‘obeamatters’ throughout the archipelago. Given that it was difficult to prove actual Obeah acts, the possession of such objects often led to convictions and punishment on other islands.^38^ The Swedish Lutheran priest Johan Forsström—who had qualified as a medical doctor before he had taken up theology—examined the objects. He reported that they included a turtle egg and that the umbilical cord was actually the intestine of some small animal. He added that the linen in the bundle could not have been buried in the ground for more than eight days. Lion claimed the yard had been levelled two years earlier and had not been touched thereafter.

Forsström’s and Lion’s testimonies are not the only contradictions in these proceedings. Many different interpretations could be made, the most obvious one that Rosalie by some sleight of hand had managed to hide the bundle in the earth heap beside the hole while Jean-Baptiste and Gabriel were digging. It is worth bearing in mind Yvan Debbasch’s comment that when discussing poisoning in the Antilles it is sometimes useless to ‘trace a neat border between truth and illusion’ as we cannot establish the facts.^39^

## Reprimanding ‘Crafty Arts’

Upon completing their deliberations, the court reprimanded Bernard Lion for using the services of an ‘ignorant coastal negro’ and letting himself be fooled by Rosalie’s ‘crafty arts’.^40^ Lion was told to henceforth use the services of the island’s licensed physicians. Lion, who owned a store and smaller ships, was at least partially illiterate; he signed his petitions with a cross. The fact that he was Catholic might have influenced the court to criticise his resorting to what could be constructed as the ‘papist delusions’ King Gustav had lambasted when decriminalising witchcraft in the metropole. Though freedom of religion was declared on the island the administrators were at times sceptical of Catholic religious expressions.^41^

The court had both Jean-Baptiste’s confession and witness accounts that he had practised medicine for a long time. But, as there was no proof that Jean-Baptiste or Gabriel had caused any bodily harm they were acquitted. The court threatened Jean-Baptiste with a so-called extrajudicial punishment—usually 29 whiplashes—if he were to occupy himself with ‘potion-mixing and medical treatments’ again.^42^

According to the court, Rosalie’s worst crime was that she had made false accusations against Jean-Baptiste and Gabriel. The gravity of the accusations suggests they were engendered by rivalry, just like the rivalry amongst the group of white physicians who often competed for patients and government assignments. Another issue at stake may well have been their different origins. Jean-Baptiste had introduced new knowledge from Africa, possibly giving him a reputational advantage in comparison to Rosalie, who was a native of the Americas.

The court also felt that Rosalie’s deception of Lion by ‘tricks along with superstitious and deceitful measures’ deserved punishment, but as Lion had paid Rosalie’s owner a high fee in security, the court ordered him to immediately send her back to St Martin.^43^ Rosalie was admonished never to repeat her ‘tricks and supposed supernatural arts’ in St Barthélemy as next time she would be punished.^44^

By transforming ‘magic’ into ‘supposed supernatural arts’ the court avoided punishing practices that were common throughout the archipelago. An argument that has been put forward is that the often European-born members of Caribbean courts did not understand references to Obeah and magic in the cases they presided over.^45^ This might, of course, often have been the case, but it is also possible that courts intentionally avoided dealing with ‘magical’ elements as it would complicate proceedings. Recognising such elements would also require a deeper understanding of the shared belief systems of the enslaved population and the white people who used their services. That judicial officials were well aware of the influence of Obeah is exemplified by Erik Olof Bergius, who served as judge on St Barthélemy 1813–1816. He wrote how ‘poisoning, along with other pretended magic, is executed in the greatest secrecy by old negresses under the name Obia, and is so feared by the negroes, that merely the conviction of being the object of such magic has caused many to waste away and die’.^46^

This and several of the following cases prove that the Black healers and their activities were tolerated. The intention here is not to portray Sweden as a less cruel colonizer than other European powers, a trope that is for instance still present in the historiography on the Swedish seventeenth century Delaware territory.^47^ However, the court’s lenient treatment of Black healers, until it was proven they had committed some other worse crime that threatened social order, was a recurring aspect of the Swedish colonial justice system.^48^

## Shared Beliefs and Practices

The proceedings against Jean-Baptiste and Rosalie clarify the widespread sharing, borrowing and incorporation of practices and beliefs in the developing culture of healing in St Barthélemy. The Swedish Government Doctor Samuel Fahlberg was a member of the court that tried the case.^49^ He was a dedicated naturalist and especially interested in medicinal plants. He published on remedies favoured by Black inhabitants: ‘A potion is prepared with the bark [Zanthoxylum fagara] and used against venereal scabies and its wounds. The negroes drink when required half a quart every morning’. The sap of Plumeria alba ‘is used by the negroes on fresh wounds .. Women use this sap as an emmenagogue [and it is] also utilised by lecherous persons as it is believed to be a potent abortifacient’.^50^ Fahlberg had great knowledge of the local pharmacopoeia and sometimes experimented on himself. He probably concluded that Jean-Baptiste and his colleagues did no harm. Fahlberg also stressed the cross-community sharing of beliefs in both the medical and magical properties of remedies: ‘Many superstitious whites, and especially negroes, around Christmas and New Year’s daub their clothes on the back, chest and arms, with points, lines and crosses with the juice of this fruit [Opuntia ficus-indica], and believe thus they will be particularly fortunate in all their endeavours all through the year’.^51^

Just as European medical practitioners often showed interest in the knowledge of Black herbalists, Black healers also turned towards European practices. The example of the enslaved surgeon Coq d’Inde, who bled his patients, indicates the exchange of medical methods and knowledge. How medical knowledge was created and transferred in the Caribbean is the object of much research and it bears remembering that investigating this transfer is not a new field.^52^ The British 1788 parliamentary inquiry into the slave trade asked a question concerning medical practices: ‘Are these Arts or Means brought by the Obeah-men from Africa, or are they Inventions which have originated in the Island[s]?’^53^

Knowledge transfer between white and Black practitioners was also conditioned by market forces. Economic considerations drove white physicians to disregard local remedies, as a French author observed in 1788: ‘The young persons who come here [to the Antilles from Europe] to practise medicine and surgery are too seduced by the love of profit, they could, instead, more usefully dedicate themselves to the study of the simples in which this country abounds without harming their fortunes.’^54^

## Understanding Black Healing

By shaming Bernard Lion—and the mocking articles in the newspaper edited by the judge had the same effect—the court signalled to the white population that enslaved healers should be avoided, and that licensed doctors be consulted instead. This was also probably meant to protect white physicians’ businesses.

Today it is clear that the treatments offered by white physicians were often more damaging than the remedies prescribed by Black healers. Richard Sheridan put it succinctly: ‘Black slave medics . . . were often more effective in their cures than their white counterparts … Rather than routinely purging, puking, and bleeding their patients, the black doctors administered herbs and roots that frequently contained curative properties. Even if their remedies did not cure, they did not kill as did opium, mercury, antimony, and venesection’.^55^ Some early colonisers drew similar conclusions.^56^ The British agent for Grenada and St Christopher sang their praises: ‘From their skill in Simples, and the Virtues of plants, they sometimes operate extraordinary Cures in Diseases which have baffled the Skill of regular Practitioners … I have myself made use of their Skill for the last with great Success’.^57^

This attitude might have inspired the St Barthélemy court of justice. It did not consider the Black healers’ medical services physically harmful. Indeed, Fahlberg might even have believed them beneficial. The court refrained from punishing the Black healers for administering medicine, something that in a Caribbean context could have been interpreted as legal condoning of their activities. In addition, the court regarded their magical activities as mere fraud; thus evidencing an approach to Black healing that foreshadows the development in, for instance, the post-emancipation British Caribbean.^58^

Such a distinction between the administration of herbal remedies and the enactment of supposedly supernatural feats may in itself have been misguided. Investigations into African Caribbean religious practices have shown that medicinal, and what appeared to the Swedish officials to be magical, practices were often part of the same belief systems. White inhabitants who trusted Black healers probably understood this better than the members of the court.

The Swedish court expressed its bafflement in the ‘peculiar case’ against Rosalie and Jean-Baptiste.^59^ The case highlights how the magical elements of Black healing fell outside of the Swedish officials’ understanding of medicine.

## Protection and Poison

The case against Bernard in 1800 illustrates the Swedish determination to undermine the credibility of Black healers. Bernard was accused by several militia members of being a ‘conjuror and a so-called poisoner’ and resisted arrest and was injured.^60^ Once put in irons he threatened the soldiers that if they did not set him free the governor would have an accident and the soldiers would suffer misfortune. Bernard claimed that no iron bars were strong enough to jail him and promised to take revenge on his captors.

Bernard was from Dominica and had been in St Barthélemy for a couple of years. He was known as a seller of small pouches. These were inspected by the court and found to contain ‘innocuous barks and roots’.^61^ An informant told the court that the pouches Bernard sold offered protection against being captured at sea. The island’s military commander pleaded with the court to prosecute Bernard ‘to prevent the effects that the coloured commonly believe such a chatterbox is capable of generating’.^62^ Although the case went to trial no records of the sentence survive. As in the Bernard Lion case, the authorities’ professed aim was to stem the perceived deleterious effects of superstition among the population.

Black healers were not only taken to court for trying to cure maladies or spreading superstition; they were also accused of selling poison. In February 1808, the merchant Joseph Cremony found his slave Romeo tied up in his yard.^63^ Several of Cremony’s slaves accused Romeo of poisoning Atis—alias Petit Congo—who had died a few days earlier. According to the slaves Romeo’s motive for doing this was that he was in love with Atis’ wife. They told Cremony that if he did not have Romeo jailed, they would drown him in the sea. Cremony obeyed and the court initiated a thorough investigation.

It started by consulting Louis Toussaint Paris, the surgeon who had treated Atis, a heavy drinker, for recurring stomach problems. Paris’s autopsy report concluded that ‘the main cause of death is found in the notable disturbance of the stomach and the intestines, caused by a poison that I believe is a juice of a herbaceous and vegetal nature’.^64^ Paris, who was born in France, had previously worked on Saint-Domingue and as his licence to practise in St Barthélemy was granted in 1805 it is probable that he left Saint-Domingue during the war that ended with Haitian independence in 1804.^65^ Perhaps it was his Saint-Dominguian experiences that made him conclude with such assertion that the cause of death was poison. Both before and during the Haitian Revolution the white population had especially feared both poisoning and magical practices.^66^

The case ran for several court sessions and contradictory testimonies added to the confusion. According to several of Cremony’s slaves it was Jean-Baptiste—who had been acquitted less than two years earlier—who had provided the poison that Romeo had added to Atis’ rum. Paris’s report that Atis was poisoned would likely have resulted in a severe punishment for both the imputed poisoner and the alleged poison provider in a French Caribbean jurisdiction.

In the Rosalie case Jean-Baptiste had claimed only to administer medicine, but he was now accused of providing poison. Considering Jean-Baptiste’s fame as a healer and Atis’ record of health problems, it is not impossible that Jean-Baptiste had treated Atis and thus was also a direct rival of Paris. The step from accusing Black healers of administering a ‘herbaceous juice’ as a treatment, to providing poison of likewise vegetal origins was a conceptual leap that had been undertaken by the white population in the Caribbean for centuries.^67^ Judge Bergstedt speculated in the deliberations that the rumours about poisoning had started with the autopsy report. He thus proposed that the accusations originating from the Black community were inspired by Paris’s conviction that Atis was murdered by poison. The fact that many of the slaves who accused Jean-Baptiste were born in Africa might have influenced Bergstedt’s reasoning. He suggested that the white surgeon’s belief in poisoning had sparked a similar understanding of the events by the slaves who accused Romeo and Jean-Baptiste.

However, the judge concluded that Paris’s autopsy results were not ‘completely infallible’ and did not constitute sufficient proof of poisoning.^68^ Even if Atis had been poisoned, it was impossible to sentence anyone without a confession or credible witnesses. The alleged involvement of Jean-Baptiste as the poison provider did not lead to further investigations, despite the court in the Lion case having threatened to punish him if he ever relapsed. The case was dismissed and the jailed absolved of any offences.

This and other ‘poisoning’ cases treated here were brought to the authorities’ attention by island inhabitants. The outcomes of this and Bernard Lion’s case signalled the court’s intention to eradicate ‘prejudices and superstition’ in the field of healing. This message was not only directed towards ‘less enlightened persons’ like Lion but also towards the educated surgeon Paris, as well as towards the Black healers.

## Dangers of Magical Services

Notwithstanding the reluctance to severely punish Black healers in the previous cases, there was nothing benevolent about judicial practice in St Barthélemy. This is evident in the case where the young enslaved woman Daly (c. 25 years old) strove to be manumitted with the help of potions. Common crimes perceived to threaten the social order and committed by the enslaved population were harshly punished.^69^

That healers’ services could be dangerous for the purchaser is not uncommon, and in the case of Daly and her business dealings with the enslaved woman Sally it is a challenge to view the role of the healer in positive terms.^70^

In 1811, Sally convinced Daly to steal a substantial sum of money from her owner, Customs Master Anders Furuträd. The theft was discovered and Daly confessed, telling the court that she had given most of the stolen money to Sally who had promised to make Daly’s employer fall in love with her and then manumit her. Sally had prepared a ‘love powder’, allegedly out of ground human bones from the town’s cemetery, which Daly was instructed to mix with Furuträd’s liquor.^71^ Sally concocted the following potion with Furuträd’s hair and old clothes, and gave Daly objects to put in Furuträd’s pillow. She continuously increased her demands and Daly stole additional money to pay for ever more complicated formulas. Daly claimed she had paid Sally enough money for her to buy her own freedom as well as to acquire two slaves. This turned out to be true. When Sally’s former owner was questioned why she had not inquired as to the origins of the money with which Sally bought her freedom, she told the court that no such thing had occurred. Instead an Italian mariner had insisted on buying Sally, who thus had convinced the Italian to buy her for her stolen money so that she could then be freed on another island without raising suspicions. By the time Furuträd noticed the missing money, Sally had already left the island. If apprehended she would have been whipped, sold back into slavery and banished.

Daly desperately wanted to be manumitted herself, but like Sally she understood that if she had used the money to try to buy her freedom, her theft would have been immediately discovered. Daly also bought items with the stolen money that she sent to be sold on other islands. Several slaves were accused of receiving stolen goods but as it could not be proven that they knew about Daly’s thefts they were acquitted. Maybe Daly had learnt from Sally and tried to launder money in the adjacent colonies.

Daly was eventually sentenced after 13 months in jail. She was sick and pleaded for mercy. The court instructed the Government Doctor Jacob Leurén to examine her and determine whether she was healthy enough to endure physical punishment. Except for ‘hysterical attacks’ and digestive problems due to the incarceration he did not find any ‘major corporal weakness’ that impeded her punishment.^72^ The court ordered her to be immediately whipped and sold off the island.

Daly’s case acts as a corrective to the sometimes romanticised view of the Black healer as a mainly supportive figure. However, it is also possible that Sally believed in her cures and thought that they would make Furuträd fall in love with Daly, at the same time as she convinced Daly to commit further thefts. That Daly trusted the potions to be efficient, or was despondent enough to try every possible way to get manumitted, is also proven by the fact that she contracted another enslaved healer after Sally’s departure. Daly paid the woman Monimia to prepare new potions that she mixed in Furuträd’s food and drink.

Despite the actions of women like Sally and Rosalie, who offered magical services that can be interpreted as deleterious to other Black persons, there was a significant number of Black women who worked as what might today be more easily defined as healers, and several of these women were midwives.^73^

## Midwives and Female Apothecaries

The sharing of and overlaps between Black and white healing methods stand out with regard to the island’s enslaved midwives. The Swedish administrators allowed these women to continue their work with minimal interventions. Doctor Fahlberg confirmed that enslaved midwives were the norm on St Barthélemy when he was interrogated in a court case regarding the mistreatment of a newborn child. He vouched for the midwife who had delivered the baby. She was the ‘only one on this island who he could recommend in the capacity of a midwife, and that the said Catherine had delivered his own wife of a child in the year 1791’.^74^ That white women—including Fahlberg’s wife—preferred Black midwives was common in the Antilles, as a Saint-Domingue doctor noted the same year: ‘There are always women of colour in the cities who are trained to deliver babies, and we often notice that white women prefer them to midwives’.^75^ It was never proposed that St Barthélemy’s midwives be licensed according to Swedish standards, which demanded literacy and apprenticeship. Such requirements would have forced local midwives to practise illegally and decreased the availability of their services. By leaving the area unregulated, these women remained important figures in the field of healing, offering their services to both white and Black clients.^76^

But there were limits to government’s acceptance of the behaviour of the island’s enslaved midwives. In 1792, a rumour reached the court that the free Black woman Rachel Goodman had miscarried. Fahlberg examined her and concluded that she had not been pregnant recently; another doctor confirmed this.

Rachel was in a relationship with Count Olof Wilhelm Leijonstedt, a Swedish officer exiled to the island for treason following his opposition to the 1788–1790 Russo-Swedish war. It turned out that Rachel, with the collusion of the enslaved midwife Elizabeth, had tried to convince Leijonstedt that she had given birth to a stillborn boy.^77^ Elizabeth admitted in court that Rachel had bled some but that she had not given birth. She also confessed to starting the rumour that Rachel had given birth on Rachel’s instigation, and that she had buried some bloody pieces of cloth in front of the Catholic Church’s presbytery, the building where Rachel Goodman and Leijonstedt lived together. The burial place of the cloth was probably connected to spiritual practices. The burying of objects in particular places, for instance at thresholds and in front of doors, was often connected to Black Atlantic rituals.^78^ It was not a coincidence that Rosalie had ordered Jean-Baptiste and Gabriel to dig close to the gate of Lion’s yard.

Fahlberg had the bloody pieces of cloth dug up. The court informed the midwife Elizabeth that she would be severely punished if she ever spread such rumours again. By supporting Rachel Goodman’s attempt to curry favour with Leijonstedt Elizabeth had acted against the interest of white inhabitants, and this was not acceptable.

Fahlberg ordered the bloody textiles to be buried as far away as possible from the ‘cemetery and the church’s hallowed ground’.^79^ The court explicitly recognised that the burial place of the bloody pieces of cloth mattered on the grounds of European spiritual convictions, thus acknowledging that the Lutheran Swedes also assigned religious significance to specific places.

In addition to midwives, there were also female Black apothecaries who treated ‘common negro illnesses’ in St Barthélemy.^80^ Nancy Davis, a free Black woman from St Martin, briefly ended up in jail in 1808. It is not clear whether she had been called over from St Martin on the grounds of her medical knowledge, but she admitted to treating several slaves and during the court proceedings it again emerged that the slave owners had requested and paid for her services. Davis had recommended salt water baths and used creams to treat Daba’s and Blackstan’s joint pains and Mary’s eye condition. None of the slaves were given any medicine to ingest according to witnesses. The court referred to Article 6 in the 1787 law and fined Davis the stipulated 500 livres (approximately 56 Spanish dollars). It also stated that she was destitute and that the fine would normally have been commuted to a prison sentence on bread and water. As the government would have to bear the costs of her imprisonment, she was instead freed and ordered to leave the island within eight days never to return. Black people were indeed kept in jail at the government’s expense for minor offences but Nancy Davis’s crime was considered of so little importance that she did not need to be made an example of. The case is also a sign of the increasing social importance of the free Black population in both St Barthélemy and the surrounding islands, something which the career of another apothecary also made evident.

## Licensing Black Surgeons

The timeframe of the cases presented here is around thirty years and this period was tumultuous even by Caribbean standards. From the sparsely populated island the Swedes had found upon arrival in 1785, St Barthélemy became an important marketplace in the 1810s. This is mirrored in the developments in healing and medicine on the island. What had once only been offered by one creole slave—Coq d’Inde—had been replaced by a multitude of both enslaved and free Black healers offering their services in St Barthélemy and the adjacent colonies.

The same year (1808) that Nancy Davis was sentenced a free Black man named James Wattley was prosecuted for similar crimes. Wattley’s rise in social standing as a medical practitioner is suggestive of the changes that led to opportunities for a free Black person that would have been unimaginable at the time of the Swedish acquisition of the colony.

At his first court appearance in 1808, he defended himself by claiming that there were several other non-licensed sellers of medicine in Gustavia.^81^ Witnesses confirmed that he had drained abscesses and dispensed medicine, in one case consisting of ‘bark, a dose of Rhubarb and some salt’. Vice-Fiscal David Ludvig Falkman referred to the 1787 law and the 1800 regulation requiring permission to practise medicine, as well as metropolitan laws regulating the sale of medicaments. Wattley had treated ‘less educated people’, a locution usually applied to the poorer white population.^82^ He was fined 16 Spanish dollars instead of the stipulated 56 and threatened with banishment if he relapsed.

Five years later in 1813, it was instead Wattley who turned to the authorities and applied for a licence to practise medicine. He presented several certificates proving that during the intervening years he had been a surgeon’s apprentice on the neighbouring island of St Kitts as well as with a doctor in St Barthélemy. He had also worked as a surgeon aboard one of the Swedish merchant Adolf Fredrik Hansen’s slave ships. Captain Miguel Nuñez of the *Brillante Rosa* attested that Wattley had saved a man’s life by amputating his arm at sea off the Angolan coast in 1811.^83^ Wattley is yet another example of how mobile St Barthélemy’s Black healers were: not only had he trained on several islands but he had also practised his profession in Africa.

Government Doctor Leurén evaluated Wattley’s application and pointed out that he had no formal education. Leurén was often negative towards licensing new doctors. The next year a white man educated in Connecticut was refused a license on the grounds that there was already a sufficient number of physicians on the island.^84^

Nevertheless, on the basis of the documentation and Leurén’s own experience of Wattley’s knowledge he recommended that Wattley should be granted permission to practise as a surgeon [fältskär] and have the right to dispense medicine. Leurén also stated that Wattley predominately served the Black population, confirmed in the following years’ court protocols. In 1813 Wattley became the first Black person to lawfully practise medicine in the colony.^85^

If interpreted benignly, permitting Wattley to practise was aimed at facilitating the poor inhabitants’ access to government-approved medical services. A licensed Black surgeon who charged lower prices than white physicians reduced the need to seek the help of unlicensed healers and would not have infringed much on the white doctors’ income. What can be safely concluded is that the licensing of Wattley—unthinkable just a few decades earlier—is one of several developments that indicate the increasing importance of the colony’s free Black population and how its conditions were slowly improving.

## Conclusion

This outline of Black healing on St Barthélemy bears out several important, and also suggestive, insights into the role of Black participation in medicine, magic and knowledge on the Swedish island and beyond. Though often overlooked in attempts to get deeper into the meaning of Obeah/healing, it is clear that many healers operated beyond single island shores and that this mobility was at the core of how these practices developed and spread. Coq d’Inde and Charles were already on the island before the Swedes arrived while Jean-Baptiste had been captured in Africa, Rosalie was born in Guyana, Bernard was from Dominica, Gabriel from Guadeloupe, Nancy Davis from St Martin and James Wattley had worked on St Kitts and on slave ships. The common practice of banishment served as an unintended yet efficient way of spreading knowledge and practices. Sally wisely left the island using Daly’s stolen money to be manumitted elsewhere and may have continued to practise as a free woman on other islands.

Following the French Revolution and the rebellions on Martinique and Saint-Domingue war reigned throughout the region and many islands changed hands during the period. St Barthélemy received several waves of immigrants during these decades, including Doctor Paris, who arrived from newly independent Haiti. The island’s highly mobile population is an example of the necessity of formulating a more expansive archipelagic context to allow proper investigation into how medical knowledge and practises were disseminated.

The Swedish court avoided punishing healers brought up on charges even though they often had broken the law. Several reasons for this leniency has been suggested, a major factor being the Swedes’ late arrival in the Caribbean and the subsequent lack of historical association between Obeah and rebellion.

The Black healers’ cures were rather understood in a context of quackery, and even if they were not regarded as beneficial, they did not appear to do any great harm. Several of the cases show how the court believed that what it considered fraud was a more serious crime than the actual medical work.

An additional aspect may have been that the court’s professed aim to eradicate ‘prejudices and superstition’ was a rather peripheral issue in the greater judicial context where enslaved who had committed for instance theft were dreadfully punished.

This stance was obviously understood by the healers as they carried on their activities. In some of the cases, members of the Black population used—exactly as white inhabitants did—the Swedish court to settle conflicts within their community.

The association between the healers’ activities and rebellion still serve as a constituent factor in the common interpretative frame which has reduced the activities of Black healers to acts of resistance.^86^ In some cases, this might be a valid perspective. The protective pouches Bernard sold may, for instance, have made enslaved sailors more prone to escape their owners. However, by interpreting the Black healers’ activities mainly in a framework of resistance we might perpetuate the white population’s fear of Black rebellion instead of capturing other facets of the healers’ importance. By letting the white population’s prejudices influence our interpretation of the Black healers’ activities we also risk justifying the repression of their work by colonial authorities.

Diana Paton underlines that ‘[o]beah then, even during slavery, cannot be explained entirely in relation to resistance, rebellion, or even healing; it was also entangled in complex interpersonal relationships that could cross boundaries between slave and free’.^87^ Indeed, both Blacks and whites trusted the abilities of Black healers in St Barthélemy, just as they did in many other places in the Caribbean.

After having surveyed several decades of St Barthélemy court records, I have the impression that divisions in the Swedish colony were not only defined by skin colour or the distinction between enslaved and free, but often also to a large extent by language and geographical origin, faith, class and kinship bonds.

In parallel to the attempts to identify magic and poisoning as expressions of resistance, the St Barthélemy healers illustrate a different type of agency. Several of them were health entrepreneurs of sorts, and irrespective of their status they worked for money, attending to the needs of both Black and white communities. They moved throughout the Caribbean, through compulsion and free choice, and serve as examples of an archipelagic history which extends beyond the strictures of nation state and single island narratives.

## Footnotes

1 The terms used to name the enslaved and free population of African descent in the Caribbean have varied over time. I use the capitalised Black as it encompasses many groups that have formerly been distinguished by various terms. In the same vein, I use the general term healer as it has the advantage of connoting both the physical and spiritual services requested by the clientele. The literature on Caribbean medicine and magic has grown substantially in recent decades. Useful surveys are found in: Diana Paton, *The Cultural Politics of Obeah: Religion, Colonialism and Modernity in the Caribbean World* (Cambridge: Cambridge University Press, 2015); Caroline Oudin-Bastide, *L’effroi et la terreur: esclavage, poison et sorcellerie aux Antilles* (Paris: la Découverte, 2013).

2 All translations are mine. Guadeloupe Governor Baron de Clugny, État de l’isle St. Barthélemy au commencement de 1785, fol. 206, F/3/54, Archives nationales d’outre-mer (henceforth ANOM), Aix-en-Provence, France: ‘un nègre Esclave sert de médecin dans l’isle, il a la confiance de tout le monde, ou le dit fort entendu pour la saignée, les fractures et blessures’. Dénombrement du vent, 13 February 1787, vol. 28, S:t Barthélemysamlingen, Swedish National Archives [Riksarkivet], Stockholm, Sweden.

3 Governor Rayalin’s order book, 15 May 1785, vol. 325, Fonds suédois de Saint-Barthélemy (henceforth FSB), ANOM: ‘de la part de tous les habitants’; ‘Nègre nommé Charles … est un empoisonneur … ne le permettant pas de rester dans l’isle, voulant prévenir des accident fâcheux qui peuvent arrivés par un ainsi méchant homme.’

4 Proceedings against slaves were often held with minimal record keeping in the British colonies, while almost all French slave court archives have been destroyed, some of them intentionally, see: Marie Houllemare, ‘Vers la centralisation des archives coloniales françaises au xviii^e^ siècle: destruction et conservation des papiers judiciaires’, in Maria Pia Donato and Anne Saada, eds, *Pratiques d’archives à l’époque moderne: Europe, mondes coloniaux* (Paris: Classiques Garnier, 2019), 349–67; Diana Paton, ‘Punishment, Crime, and the Bodies of Slaves in Eighteenth-Century Jamaica’, *Journal of Social History*, 2001, 34, 923–54. On Danish court proceedings involving the Black population see: Gunvor Simonsen, *Slave Stories: Law, Representation, and Gender in the Danish West Indies* (Aarhus: Aarhus University Press, 2017). The idea of the ‘silence’ of the Black population in the archival record is being nuanced by recent investigations, for the situation on the French islands see: Dominique Rogers, ed., *Voix d’esclaves: Antilles, Guyane et Louisiane françaises, XVIIIe-XIXe siècles* (Paris: Karthala, 2015); Frédéric Régent, Gilda Gonfier and Bruno Maillard, eds, *Libres et sans fers: paroles d’esclaves français: Guadeloupe, Îles Bourbon (Réunion), Martinique* (Paris: Fayard, 2015). In the British case, see e.g.: Natalie Zacek ‘Voices and Silences: The Problem of Slave Testimony in the English West Indian Law Court’, *Slavery & Abolition*, 2003, 24, 24–39.

5 Londa Schiebinger, *Secret Cures of Slaves: People, Plants, and Medicine in the Eighteenth-Century Atlantic World* (Stanford: Stanford University Press, 2017), 164.

6 Pablo F. Gómez, ‘Incommensurable Epistemologies? The Atlantic Geography of Healing in the Early Modern Caribbean’, *Small Axe*, 2014, 18, 95–107, 97. This argument is developed in Gómez’ book: *The Experiential Caribbean: Creating Knowledge and Healing in the Early Modern Atlantic* (Chapel Hill: The University of North Carolina Press, 2017). For an insightful discussion of Gómez’ and Schiebinger’s work see Kristen Block’s review essay: ‘Science and Slavery in Historiographical Evolution,’ *Slavery & Abolition*, 2018, 39, 756–65.

7 The economic transactions between client and supplier elude us in the St Barthélemy cases, for a discussion on monetary exchange see: Maarit Forde, ‘The Moral Economy of Spiritual Work: Money and Rituals in Trinidad and Tobago’, in Diana Paton and Maarit Forde, eds, *Obeah and Other Powers: The Politics of Caribbean Religion and Healing* (Durham: Duke University Press, 2012), 198–219.

8 Recently several authors have discussed how to overcome such boundaries, see: Ernesto Bassi, *An Aqueous Territory: Sailor Geographies and New Granada’s Transimperial Greater Caribbean* (Durham: Duke University Press, 2016); Jeppe Mulich, ‘Microregionalism and Intercolonial Connections: The Case of the Danish West Indies, 1730–1830*’, Journal of Global History*, 2013, 8, 72–94.

9 Swedish Caribbean colonial historiography is underdeveloped, but is presently growing and doctoral dissertations numbers three and four (the first in 1888 and the second in 1951) on the Swedish possession were defended in 2016: Victor Wilson, *Commerce in Disguise: War and Trade in the Caribbean Free Port of Gustavia, 1793–1815* (dissertation, Åbo Akademi University, 2016); Ale Pålsson, *Our Side of the Water: Political Culture in the Swedish Colony of St Barthélemy 1800–1825* (dissertation, Stockholm University, 2016).

10 The law did not have a title when proclaimed and was often referred to as the ‘colonial law’ by the court. The title derives from a printed version in the island’s newspaper *The Report of St. Bartholomew*, no. 60, 20 July 1805. The Martiniquan original was the *Ordonnance du gouvernement, concernant la Police générale des Nègres & Gens de couleur libres*, 25 December 1783, first printed in Jacques Petit de Viévigne, ed., *Second supplément au Code de la Martinique* (Saint-Pierre, Martinique: Pierre Richard, 1786), 324–34. This ordinance was important in the evolution of French Caribbean slave law, see: Jean-François Niort, ‘De l’ordonnance royale de mars 1685 à l’ordonnance locale sur la police générale des Nègres de décembre 1783: remarques sur le “Code Noir” et son évolution juridique aux iles françaises du vent sous l’ancien régime’, *Bulletin de la société d’histoire de la Guadeloupe*, 2016, no. 173, 37–52.

11 This and next quote from the MS version in English, 30 June 1787, vol. 134, FSB.

12 Viévigne, *Second supplément*, 326: ‘pour arrêter un désordre qui ruine plusieurs habitans’.

13 For an overview of Swedish Caribbean slave law and judicial practice see: Fredrik Thomasson, ‘Thirty-Two Lashes at Quatre Piquets: Slave Laws and Justice in the Swedish Colony of St. Barthélemy ca. 1800’, in Holger Weiss, ed., *Ports of Globalisation, Places of Creolisation: Nordic Possessions in the Atlantic World during the Era of the Slave Trade* (Leiden: Brill, 2016), 280–305.

14 See e.g.: Jerome S. Handler and Kenneth M. Bilby, ‘On the Early Use and Origin of the Term “Obeah” in Barbados and the Anglophone Caribbean’, *Slavery & Abolition*, 2001, 22, 87–100; Oudin-Bastide, *L’effroi et la terreur*.

15 Diana Paton, ‘Witchcraft, Poison, Law, and Atlantic Slavery’, *William and Mary Quarterly*, 2012, 3rd ser., 69, 235–64; Paton, *The Cultural Politics of Obeah*; Oudin-Bastide, *L’effroi et la terreur*, 17. For the effects of the intermixing of judicial systems on the treatment of poisoning, witchcraft and obeah in Trinidad see: James Epstein, *Scandal of Colonial Rule: Power and Subversion in the British Atlantic during the Age of Revolution* (Cambridge: Cambridge University Press, 2012),

16 The first French medical regulation concerning the Black population was proclaimed in 1674: Louis Joubert, *Histoire et législation de la pharmacie aux Antilles françaises* (dissertation, Université de Strasbourg, 1955), 23; an overview of French legislation and policing of ‘poisoning’ in: Hurard Bellance, *La police des noirs en Amérique (Martinique, Guadeloupe, Guyane, Saint-Domingue) et en France aux XVIIe et XVIIIe siècles* (Matoury: Ibis Rouge, 2011), 121–53; for the development of legislation in Saint-Domingue/Haiti: Kate Ramsey, *The Spirits and the Law: Vodou and Power in Haiti* (Chicago: University of Chicago Press, 2011); in the British Caribbean: Jerome S. Handler and Kenneth M. Bilby, *Enacting Power: The Criminalization of Obeah in the Anglophone Caribbean, 1760–2011* (Kingston, Jamaica: University of the West Indies Press, 2012); in the Dutch Caribbean: Bastiaan D. van der Velden, *Ik lach met Grotius, en alle die prullen van boeken: Een rechtsgeschiedenis van Curaçao* (Willemstad: CARIB, 2011), esp. ch. 15; and for Danish Caribbean legislation: Gunvor Simonsen, ‘Magic, Obeah and Law in the Danish West Indies, 1750s–1840s’, in Weiss, *Ports of Globalisation*, 245–79; Gunvor Simonsen, ‘Risking Obeah: A Spiritual Infrastructure in the Danish West Indies, c. 1800–1848’, in Markku Hokkanen and Kalle Kananoja, eds, *Healers and Empires in Global History: Healing as Hybrid and Contested Knowledge* (Cham: Palgrave Macmillan, 2019), 203–37.

17 Jonathan Dalby, ‘Obeah in Jamaica since 1760: Criminalization, Representation, Prosecution’, Staff/postgraduate seminar paper, 9 December 2016 (University of the West Indies, Mona), 10.

18 John Savage, ‘Between Colonial Fact and French Law: Slave Poisoners and the Provostial Court in Restoration-Era Martinique’, *French Historical Studies*, 2006, *29*, 565–94; John Savage, ‘“Black Magic” and White Terror: Slave Poisoning and Colonial Society in Early 19th Century Martinique’, *Journal of Social History*, 2007, 40, 635–62; Geneviève Leti, ‘L’empoisonnement aux Antilles françaises à l’époque de l’esclavage (1724–1848)’, in Philippe Hrodej, ed., *L’esclave et les plantations: de l’établissement de la servitude à son abolition. Un hommage à Pierre Pluchon* (Rennes: Presses universitaires de Rennes, 2008), 222–27.

19 Simonsen, *Magic, Obeah and Law*, 260–61.

20 Fredrik Thomasson, ‘Révoltes et résistances dans la Caraïbe scandinave’, *Revue du Philanthrope*, 2020, 9, 125–43.

21 These numbers should only be considered as indicative as population statistics during the early Swedish decades is erratic and probably underestimate the size of the population.

22 Sten Landahl, ed., *Sveriges ridderskaps och adels riksdagsprotokoll från och med år 1719*, *del 32, 1778–1779*, (Stockholm: Riksdagens tryckeriexpedition, 1982), 454: ‘ett orimmeligit brott af Papistiska inbillningar digtadt’.

23 The witch trials of especially women in seventeenth to eighteenth century Sweden have been thoroughly investigated, see e.g.: Per Sörlin, *‘Wicked arts’: Witchcraft and Magic Trials in Southern Sweden, 1635–1754* (Leiden: Brill, 1999); Linda Oja, *Varken Gud eller natur: Synen på magi i 1600- och 1700-talets Sverige* (dissertation, Uppsala University, 1999); Soili-Maria Olli, *Visioner av världen: Hädelse och djävulspakt i justitierevisionen 1680–1789* (dissertation, Umeå University, 2007).

24 See for the period around 1800: Sofia Ling, *Kärringmedicin och vetenskap: Läkare och kvacksalverianklagade i Sverige omkring 1770–1870* (dissertation, Uppsala University, 2004).

25 11 November 1800, printed in *The Report of St. Bartholomew*, no. 33, 5 January 1805.

26 This and following quotations: *The Report of St. Bartholomew*, no. 90, 16 August 1806; no. 92, 8 October 1806; no. 94, 15 November 1806.

27 13 August 1806, vol. 150, FSB.

28 Gabriel Debien, *Les esclaves aux Antilles françaises (XVIIe-XVIIIe siècles)* (Basse-Terre: Société d’histoire de la Guadeloupe; Fort-de-France: Société d’histoire de la Martinique, 1974), 8: ‘Décrire la vie des esclaves d’après ces sources est un paradoxe. Ce ne sont jamais eux qui parlent, qui témoignent, mais les gérants ou les maîtres, qui sont blancs et qui s’expriment en colons … Il a à se rappeler que ce sont des blancs qui ont la parole.’

29 Sophie White, *Voices of the Enslaved: Love, Labor, and Longing in French Louisiana* (Chapel Hill: University of North Carolina Press, 2019).

30 13 August 1806, vol. 150, FSB.

31
*Ibid*.: ‘Långt ifrån at vilja styrka mindre uplysta menniskor uti redan för mycket rotade fördommar och vidskeplighet, men fast mer den välmenta afsigt at genom en undersökning ådaga lägga det skamlösa bedrägeri å ena sidan, och den oförlåteliga lättrogenhet å andra sidan som fram alstrat närvarande ansökning.’

32
*Ibid*.: ‘de faire usage de simples malfaisantes et autres drogues’.

33
*Ibid*.: ‘wanliga sämsta slaget, med pappers klädde ramar’.

34
*Ibid*.: ‘dryckesblandare’

35
*Ibid*.: ‘vanesinnighet’. Lion would later try to abandon Catherine when she was jailed as a result of her mental illness, 1 February 1809, vol. 156, FSB.

36 13 August 1806, vol. 150, FSB: ‘som han efter negrarnes bruk altid däraf druckit sjelf med de sjuka.’

37
*Ibid*.: ‘elaka konster’.

38 Oudin-Bastide, *L’effroi et la terreur*, 39–40; Simonsen, *Magic, Obeah and Law*, 265–266; Paton, *Cultural Politics of Obeah*, 98–99.

39 Yvan Debbasch, ‘Le crime d’empoisonnement aux îles pendant la période esclavagiste’, *Revue française d’histoire d’outre-mer*, 1963, 50, 137–88, 179: ‘tracer une nette frontière entre la vérité et l’illusion’.

40 13 August 1806, vol. 150, FSB: ‘okunnig kust neger’; ‘illistiga konstgrepp’.

41 Jan Arvid Hellström, *‘… åt alla christliga förvanter …’: En undersökning av kolonial förvaltning, religionsvård och samfundsliv på S: t Barthélemy under den svenska perioden, 1784–1878* (Uppsala: Erene, 1987).

42 13 August 1806, vol. 150, FSB: ‘dryckesblandning eller läkarewård’.

43
*Ibid*.: ‘gyckleri samt vidskepeliga och bedrägeliga anstalter’.

44
*Ibid*.: ‘gyckleri och föregifna öfvernaturliga konster’.

45 Simonsen, *Magic, Obeah and Law*, 257, 259–60; Deryck Murray, ‘Three Worships, an Old Warlock and Many Lawless Forces: The Court Trial of an African Doctor who Practised “Obeah to Cure”, in Early Nineteenth Century Jamaica’, *Journal of Southern African Studies*, 2007, 33, 811–28.

46 Olof Erik Bergius, *Om Westindien* (Stockholm: Gadelius, 1819), 34–35: ‘förgiftning, jemte annan föregifven trollkonst, utöfvas i största hemlighet af gamla Negresser under namn af Obia, och har bland Negrerne sådan skräck med sig, att endast öfvertygelsen om att vara förtrollad kommit mången bland dem att aftyna och dö.’

47 Gunlög Fur, ‘Colonialism and Swedish History – Unthinkable Connections?’ in Magdalena Naum and Jonas M. Nordin, eds, *Scandinavian Colonialism and the Rise of Modernity: Small Time Agents in a Global Arena* (New York: Springer, 2013), 1–36.

48 For examples of a similar attitude in prosecuting healers in the Spanish Caribbean: Gómez, *Experiential Caribbean*, 50.

49 On Fahlberg see Saara-Maija Kontturi, ‘Reports on Encounters of Medical Cultures: Two Physicians in Sweden’s Medical and Colonial Connections in the Late Eighteenth Century’, in Hokkanen, *Healers and Empires*, 55–79.

50 Samuel Fahlberg, ‘Anmärkningar vid åtskilliga westindiska trädarter’, *Kungliga vetenskapsakademiens handlingar* [*Proceedings of the Royal Swedish Academy of Sciences*] 1793, 153–61, 184–98, 186–87: ‘Barken nyttjas til Decoct för venerisk skabb och sår af samma art. Negrarne dricka vid sådana tilfällen alla mornar ungefär et halfstop.’; ‘nyttjas af Negrerne på färska sår … Qvinfolken bruka denna saft som et emmenagogum … Nyttjas också af liderliga personer, helst det anses som et starkt abortiens.’ For a discussion on how knowledge about abortifacients spread see: Londa Schiebinger, *Plants and Empire: Colonial Bioprospecting in the Atlantic World* (Cambridge, MA: Harvard University Press, 2004), 105–49. White slave owners sometimes consulted Black healers for treatment of venereal diseases, as in the case of the Jamaican planter Thomas Thistlewood: Trevor Burnard and Richard Follett, ‘Caribbean Slavery, British Anti-Slavery, and the Cultural Politics of Venereal Disease’, *Historical Journal*, 2012, 55, 427–51, 438.

51 Samuel Fahlberg, ‘Utdrag af samlingar til natural-historien öfver ön St. Barthelemi i Vestindien’, *Kungliga vetenskapsakademiens handlingar* [*Proceedings of the Royal Swedish Academy of Sciences*], 1786, 215–40, 248–54, 249: ‘Månge vidskeplige hvite, dock egenteligen Negrerne, bruka kring Jul och Nyårs-tiden at måla sina kläder på rygg, bröst och armar, med prickar, strek och kors, af saften i denna frukt och tro sig därigenom bekomma särdeles lycka i alt deras företagande, hela året igenom.’

52 For a recent fine-grained investigation into medical knowledge creation in the Spanish seventeenth century Caribbean see: Gómez, *Experiential Caribbean.* For a discussion of Atlantic medical knowledge exchange between white and Black communities see special issue introduction and articles: Harold J. Cook and Timothy D. Walker, ‘Circulation of Medicine in the Early Modern Atlantic World’, *Social History of Medicine*, 2013, 26, 337–51. For transfer of both medicinal and magical knowledge in Dutch colonies see: Stephen Snelders, *Vrijbuiters van de heelkunde: Op zoek naar medische kennis in de tropen 1600–1800* (Amsterdam: Atlas, 2012), 151–89; Stephen Snelders, *Leprosy and Colonialism: Suriname under Dutch Rule*, 1750–1950 (Manchester: Manchester University Press, 2017), 78–92.

53 Great Britain, House of Commons, *Report of the Lords of the Committee of Council Appointed … Concerning the Present State of the Trade to Africa, and Particularly the Trade in Slaves; and Concerning the Effects and Consequences of this Trade, as Well in Africa and the West Indies …* (London, 1789), part III, question no. 26.

54 [Nicolas-Louis Bourgeois], *Voyages intéressants dans différentes colonies françaises, espagnoles, anglaises, etc…* (London: J.-F. Bastien, 1788), 458–59: ‘L’amour du gain séduit trop les jeunes-gens qui viennent ici pour exercer la médecine & la chirurgie: ils pourraient s’occuper utilement à la connaissance des simples dont ce pays abonde, sans nuire par cette étude à leurs fortune.’

55 Richard B. Sheridan, *Doctors and Slaves: A Medical and Demographic History of Slavery in the British West Indies, 1680–1834* (Cambridge: Cambridge University Press, 1985), 336.

56 For an early French testimony see: ‘Lettres du R.P. Mongin: l’évangélisation des esclaves au XVIIe siècle’, ed., Marcel Chatillon, *Bulletin de la société d’histoire de la Guadeloupe*, 1984, no. 61–62, 115–16.

57 Great Britain, *Report of the Lords*, *Part III, Grenada and St. Christopher*, A., no. 27.

58 Paton, *Cultural Politics of Obeah*, esp. ch. 4.

59 13 August 1806, vol. 150, FSB: ‘detta besynnerliga mål’.

60 21 February 1800, vol. 145, FSB: ‘besvärjare och så kallad förgiftare’.

61
*Ibid*., ‘bark och rötter oskyldiga’.

62
*Ibid*., ‘för att förekomma den wärkan som de Couleurta uti allmänheten tror det en sådan pratmakare kunna åstadkomma’.

63 17 February 1808, vol. 154, FSB.

64
*Ibid*., ‘la cause principale de sa mort se trouve dans le désordre éminent de l’esthomac et des intestin, causé par un poison que je pense être de nature d’un suc herbacées et végétal’. For similar autopsy reports, and one stating that the victim was poisoned six months prior to death see Leti, *L’empoisonnement aux Antilles*, 214.

65 26 July 1805, vol. 149, FSB.

66 Pierre Pluchon, *Vaudou, sorciers, empoisonneurs: de Saint-Domingue à Haïti* (Paris: Karthala, 1987), 117–39.

67 On the French islands parts of the planter population remained convinced that poisoning was still a common crime at least until abolition and there are also cases where slave owners claimed that slaves had killed with only magic as late as 1838. Leti, *L’empoisonnement aux Antilles*, 214; Savage*, Colonial Fact and French Law*; Savage, *Black Magic*.

68 2 March 1808, vol. 154, FSB: ‘alldeles ofelbart’.

69 Thomasson, *Thirty-Two lashes*.

70 For an analysis of the dangers of Obeah within Black communities see for example Randy M. Browne, ‘The “Bad Business” of Obeah: Power, Authority, and the Politics of Slave Culture in the British Caribbean’, *William and Mary Quarterly*, 2011, 3rd ser., 68, 451–80; Randy M. Browne, *Surviving slavery in the British Caribbean* (Philadelphia: University of Pennsylvania Press, 2017), esp. ch. 5.

71 7 August 1811, vol. 165, FSB: ‘kärlekspulvfer’.

72 18 August 1812, vol. 165, FSB: ‘anfall af hysterie’, ‘betydelig kroppsvaghet’.

73 For Caribbean colonial midwifery see: Tara Innis, ‘“Any elderly, sensible, prudent woman”: The Practice and Practitioners of Midwifery during Slavery in the British Caribbean’, in Juanita De Barros, Steven Palmer and David Right, eds, *Health and Medicine in the Circum-Caribbean, 1800–1968* (New York: Routledge, 2009), 40–53.

74 26 August 1796, St Barthélemy council protocols, privately held MS, digital photographs in the author’s collection.

75 Charles Arthaud, *Observations sur les lois, concernant la médecine et la chirurgie dans la colonie de St-Domingue, avec des vues de règlement, adressées au Comité de Salubrité de l’Assemblée nationale et à l’Assemblée coloniale* (Cap Français: Dufour de Rians, 1791), 78: ‘Il y a toujours dans les villes des femmes de couleur, exercées dans la pratique des accouchement, et nous voyons tous les jours que les femmes blanches les préfèrent aux sages-femmes’.

76 A key law was the 14 October 1777, *Reglemente För Jorde-Gummorne* [Ordinance for mid-wives]. The degree to what extent colonial governments were interested in providing health services to the enslaved is debated, for the Danish islands see: Niklas Thode Jensen, *For the Health of the Enslaved: Slaves, Medicine and Power in the Danish West Indies, 1803–1848* (Copenhagen: Museum Tusculanum Press, 2012).

77 15–25 October 1792, St Barthélemy council protocols, privately held MS, digital photographs in the author’s collection.

78 For Caribbean examples see several contributions in Akinwumi Ogundiran and Paula Saunders, eds, *Materialities of Ritual in the Black Atlantic* (Bloomington: Indiana University Press, 2014).

79 15–25 October 1792, St Barthélemy council protocols, privately held MS, digital photographs in the author’s collection: ‘terre sainte du cimetière de l’église’.

80 Citation from case against Nancy Davis, 3 February 1808, vol. 154, FSB.

81 For other non-licensed apothecaries, see e.g., case against William Blake, 16 July 1807, vols 152, 153, FSB.

82 16 November 1808, vol. 154, FSB: ‘mindre kunnigt Folk’.

83 20 October 1813, vol. 170, FSB; 30 October 1813, vol. 168, FSB. Nuñez’ certificate dated 4 January 1811. The *Brillante Rosa* left Africa with 464 captives but only disembarked 334 at Havana in May 1811 after a 52 days passage. The Cuban doctor Tomás Romay Chacón denounced the ship’s ‘horrosa cifra de muertos’: *Obras*, ed., José López Sánchez (Havana: Imagen Contemporanea, 2005), vol. I, 258; www.slavevoyages.org/voyage/14556/variables, last accessed 9 November 2020.

84 24 November 1814, vol. 173, FSB.

85 12 November 1813, vol. 170, FSB.

86 Karol K. Weaver, *Medical Revolutionaries: The Enslaved Healers of Eighteenth-Century Saint Domingue* (Urbana: University of Illinois Press, 2006). This interpretation is rejected by e.g. Nicola H. Götz, *Obeah – Hexerei in der Karibik – zwischen Macht und Ohnmacht* (Frankfurt am Main: Peter Lang, 1995), 118–122, 210.

87 Paton, *Cultural politics*, 109.

